# Robot-Assisted versus Laparoscopic-Assisted Gastrectomy among Gastric Cancer Patients: A Retrospective Short-Term Analysis from a Single Institution in China

**DOI:** 10.1155/2019/9059176

**Published:** 2019-10-23

**Authors:** Li-Fei Sun, Kai Liu, Xue-Shang Su, Xuan Wei, Xiao-Long Chen, Wei-Han Zhang, Xin-Zu Chen, Kun Yang, Zong-Guang Zhou, Jian-Kun Hu

**Affiliations:** ^1^Department of Gastrointestinal Surgery and Laboratory of Gastric Cancer, State Key Laboratory of Biotherapy, West China Hospital, Collaborative Innovation Center for Biotherapy, Sichuan University, Chengdu, China; ^2^West China School of Medicine, Sichuan University, Chengdu, China

## Abstract

**Background:**

The da Vinci robotic system was considered an effectively alternative treatment option for early gastric cancer patients in recent years. The aim of our study was to evaluate the safety and feasibility of robot-assisted gastrectomy in our center.

**Methods:**

This study included 33 patients who underwent robot-assisted gastrectomy (RAG) and 88 patients who underwent laparoscopic-assisted gastrectomy (LAG) between January 2016 and April 2018. Clinicopathological characteristics, surgical parameters, postoperative recovery, and the learning curves of RAG were evaluated.

**Results:**

Baseline characteristics between two groups were well balanced. The operation time of RAG was longer than that of LAG (333.1 ± 61.4 min vs. 290.6 ± 39.0 min, *p* = 0.001), and the estimated blood loss was 62.4 ± 41.2 ml in the RAG group and 77.7 ± 32.3 ml in the LAG group (*p* = 0.005), respectively. The mean number of examined lymph nodes in RAG was less than that in LAG (30.3 ± 10.2 vs. 37.4 ± 13.7, *p* = 0.008). However, RAG had an advantage in the dissection of No. 9 lymph nodes (3.4 ± 2.1 vs. 2.5 ± 1.6, *p* = 0.039). The incidence of postoperative complications was similar in both groups (*p* = 0.735). There were no significant differences in terms of postoperative recovery between the two groups. The learning curve of RAG showed that the CUSUM value decreased from the 8th case, which suggested a rapid learning curve among experienced surgeons on LAG operations.

**Conclusions:**

RAG was safe and feasible for gastric cancer patients, with superiority in the dissection of No. 9 lymph nodes.

## 1. Introduction

Gastric cancer had a high incidence in Eastern Asian countries with a relative poor prognosis, especially in China. The chemotherapy resistance and distal metastasis were the reasons for high mortality of gastric cancer patients [1]. In 2015, China had an estimated 679100 patients diagnosed with gastric cancer, while 498000 patients died of gastric cancer [2]. Radical resection plus D2 lymphadenectomy was still the mainstay for the surgical treatment of gastric cancer. Considering the limitations of open surgery such as severe pain and extended incision, surgeons paid more emphasis on the minimally invasive surgery. Since the first report of laparoscopic-assisted distal gastrectomy in 1994 by Kitano et al. [3], the efficiency and innovation of laparoscopic surgery for gastric cancer had been realized. Several randomized control trials had reported their results and indicated progress about the feasibility and safety of laparoscopic surgery, such as the CLASS-01 trial for the treatment of locally advanced gastric cancer [4] and the KLASS-01 trial [5]. These studies had illuminated that patients who underwent laparoscopic-assisted gastrectomy had similar long-term oncological outcomes compared with open surgery.

However, there are some technical limitations in laparoscopic surgery, such as two-dimensional view and hand tremors. The da Vinci robotic system had overcome these limitations depending on its technical advantages; it had been applied in the surgical treatment of gastric cancer patients. Some studies had shown that robotic-assisted gastrectomy can be an alternative option for patients with comparable outcomes when compared with LAG [6–9]. Nevertheless, the high cost of robotic surgery and lack of surgical instruments were the constraints on the development of a robotic surgical system. The application of a robotic surgery system on gastric cancer patients has attracted more and more attention of clinicians in China, and studies on the safety and feasibility of RAG are becoming a focus in the region of minimally invasive surgery.

In this research, we aimed to summarize our experience and make a comparison between robot-assisted gastrectomy and laparoscopic-assisted gastrectomy by a short-term evaluation.

## 2. Methods

### 2.1. Patients

Our study retrospectively collected the data of 121 consecutive patients who underwent laparoscopic-assisted gastrectomy or robot-assisted gastrectomy in the Gastrointestinal Surgery Department, West China Hospital, Sichuan University, from January 2016 to April 2018; both types of operations were performed by the same surgeon. All the patients were informed of the advantages and disadvantages of RAG and LAG, and the informed consents were signed routinely.

**Figure 1:**
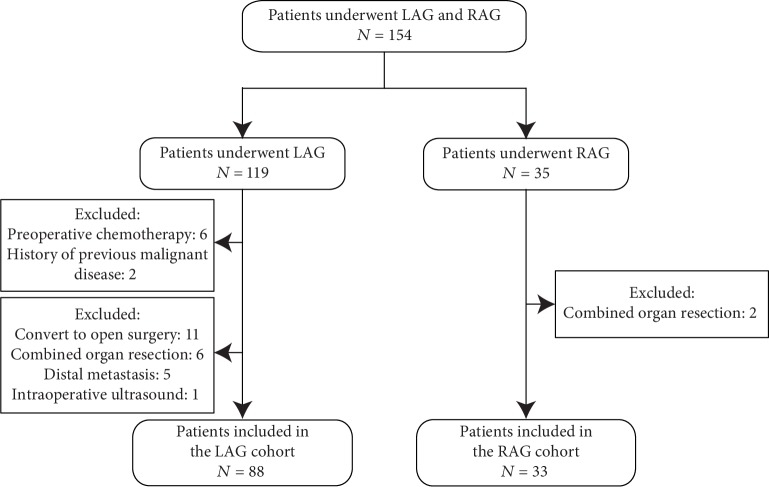
Flow chart of this study.

The inclusion criteria were the following: (1) patients who were diagnosed with primary gastric adenocarcinoma that is confirmed by an upper digestive tract endoscopic biopsy, (2) patients with R0 resection, (3) patients without any preoperative treatment, and (4) patients who underwent LAG or RAG. The exclusion criteria included the following: (1) patients with any synchronous malignancies or history of previous malignant disease, (2) patients who had received preoperative chemotherapy or radiotherapy, (3) patients who converted to open surgery, (4) patients with intraoperative ultrasound or combined organ resection, and (5) patients with distal metastasis. The inclusion and exclusion processes are shown in Figure 1.

Generally, preoperative staging was conducted through abdominal contrast-enhanced computed tomography and contrast-enhanced ultrasonography. The clinicopathological data and the postoperative pathological staging were recorded according to the Japanese classification of gastric carcinoma: 3rd English edition [10].

### 2.2. Indication

In this research, the indication for LAG and RAG referred to the “Expert consensus on quality control of the laparoscopic radical resection for gastric cancer in China (2017 edition)” and “Expert consensus on robotic surgery in gastric cancer (2015 edition)” in China [11, 12], which had indicated that LAG and RAG could be recommended for gastric cancer patients with cT1-cT3, while operation for cT4a patients belongs to exploratory indication in our clinical work. Owing to the learning curve of robotic surgery, the RAG cohort in this research included patients with a relatively early stage in order to ensure the quality of operation.

### 2.3. Surgical Procedure

According to the Japanese gastric cancer treatment guidelines [13], total or distal gastrectomy was selected according to the tumor location and D1 or D1+ lymphadenectomy was selectively implemented in patients with a relatively early stage. The da Vinci robotic platform used in this research was the da Vinci Si. The overall procedure of robotic-assisted gastrectomy is similar to that of laparoscopic-assisted gastrectomy; meanwhile, we use the clockwise modularized lymphadenectomy as previously described [14]. In terms of the digestive tract reconstruction, Roux-en-Y esophagojejunostomy was employed in patients with total gastrectomy, while the Billroth I gastroduodenostomy and Billroth II and Roux-en-Y gastrojejunostomy were adopted for distal gastrectomy.

### 2.4. Surgical and Postoperative Outcomes

The following parameters were reviewed to analyze the feasibility and safety of RAG and LAG: operation time (OT), estimated blood loss (EBL), intraoperative complications, and postoperative recovery parameters including days of the first passing flatus, hospital stay after surgery, and 30-day readmission after surgery.

### 2.5. Cumulative Sum Analysis

The cumulative sum (CUSUM) method was used to evaluate the learning curve of robotic surgery. For single-dimension CUSUM, the following equation was applied to calculate the CUSUM value: CUSUM_*n*_ = *X*_*n*_ − *μ* + CUSUM_*n*−1_, where *X*_*n*_ represented the operation time of each case while *μ* was the mean of operation time of the entire cohort. In this equation, CUSUM_0_ was set as 0. For multidimensional CUSUM, it is similar to previous studies [15, 16]. We set four variables to assess the learning curve compared with single-dimensional CUSUM: operation time, estimated blood loss, number of harvested No. 9 lymph nodes, and postoperative complications. All the variables had their own target value according to the corresponded mean value in our cohort, which was marked as *T*_0_, while the value of each case was marked as *T*_*n*_; the score of each variable was calculated as *S* = *T*_*n*_ − *T*_0_. When a case achieved the average of the entire cohort (less OT and EBL, more retrieved No. 9 LNs) or was without postoperative complications, the value of *T*_*n*_ was recorded as 0. When a case could not achieve the average (more OT and EBL, less retrieved No. 9 LNs) or was with postoperative complications, *T*_*n*_ would be recorded as 1. The rate of below average for OT and EBL and harvested No. 9 LNs was 42.42%, 30.30%, 57.58%, respectively, while the rate of postoperative complications was 18.18%. Therefore, the *T*_0_ value for four variables was set as 0.4242, 0.303, 0.5758, and 0.1818, respectively. The equation of multidimensional CUSUM in this study was defined as CUSUM_*n*_ = *S*_*n*_ + CUSUM_*n*−1_, and the CUSUM_0_ was also set as 0.

### 2.6. Statistical Analysis

All the data were analyzed by using SPSS 23.0 (IBM, USA). Learning curves of RAG were evaluated by GraphPad Prism 5 (GraphPad Software, USA). Data were expressed as mean ± standard deviation. Continuous variables were analyzed by using the independent sample *t*-test or rank sum test, while the chi-square test was used for categorical variables. *p* < 0.05 was considered statistically significant.

## 3. Results

### 3.1. Clinicopathological Features

The clinicopathological data is summarized in Table 1, and no difference was found in baseline characteristics. The robotic procedure included 33 patients while the LAG procedure consisted of 88 patients. There was no significant difference in resection type, anastomosis, and tumor location between the two groups. We found that the tumor size in the RAG group (2.3 ± 1.1 cm) was significantly smaller than that in the LAG group (2.9 ± 1.3 cm, *p* = 0.036). The TNM stage of RAG patients was lower than that of LAG patients, although it has no statistical significance (*p* = 0.168). For the lymphadenectomy, the number of retrieved lymph nodes was lesser (30.3 ± 10.2 vs. 37.4 ± 13.7, *p* = 0.008) in the RAG group while it has a significant advantage in the dissection of No. 9 lymph nodes (3.4 ± 2.1 vs. 2.5 ± 1.6, *p* = 0.039). However, we found no significant difference between the two groups in the dissection of suprapancreatic lymph nodes (10.9 ± 4.8 vs. 10.3 ± 4.2, *p* = 0.472).

### 3.2. Surgical Parameters

In RAG series, there were no patients who convert to LAG or OG, while there were 11 (9.24%) patients in the LAG group who convert to OG. The operation time for RAG patients was significantly longer than that for LAG patients (333.1 ± 61.4 min vs. 290.6 ± 39.0 min, *p* = 0.001). There was a significant decrease in estimated blood loss in the robotic gastrectomy group compared with the laparoscopic gastrectomy group (62.4 ± 41.2 ml vs. 77.7 ± 32.3 ml, *p* = 0.005). No patients had intraoperative complications in both groups.

### 3.3. The Postoperative Recovery Outcomes

In point of the postoperative recovery of the two surgical methods, the days of the first passing flatus were similar in both groups (4.8 ± 1.4 days vs. 4.2 ± 1.2 days, *p* = 0.053), as well as the hospital stay after surgery (8.8 ± 2.8 days vs. 8.8 ± 3.3 days, *p* = 0.579). And the incidence of postoperative complications had no significant difference between the RAG and LAG groups (18.18% vs. 13.64%, *p* = 0.735). Eleven patients had pulmonary infection; only one patient in the LAG group received surgical intervention because of intestinal obstruction. For the rate of 30-day readmission after surgery, there were two patients in the LAG group readmitted to the hospital because of pulmonary infection, who were cured through conservative therapy. Only one patient in the RAG group readmitted to the hospital had intestinal obstruction, who was also cured by conservative therapy.

### 3.4. Learning Curves of RAG

**Figure 2:**
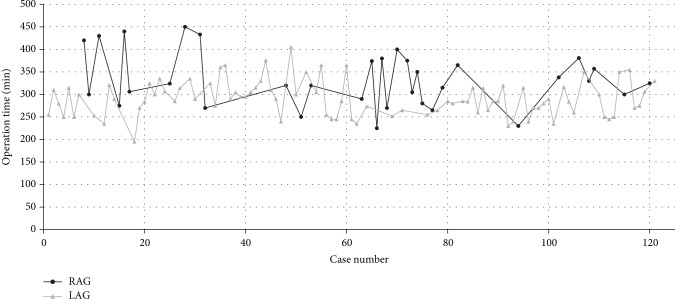
Operation time of RAG and LAG patients in the order of date of operation.

**Figure 3:**
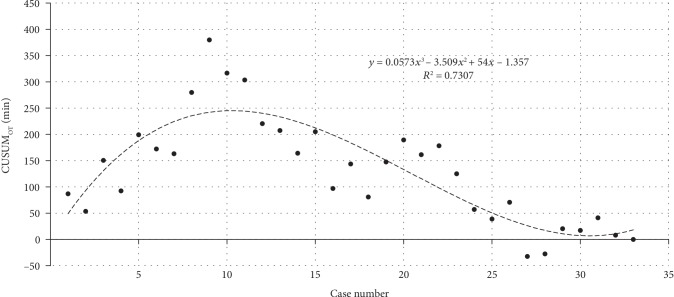
Learning curve for RAG in terms of the cumulative sum of operation time. Dashed line showed the best fitted curve; the equation was *y* = 0.0573*x*^3^–3.509*x*^2^ + 54*x*–1.357 (*R*^2^ = 0.7307), where *x* represented the case number. The cut-off value was the 10th case.

**Figure 4:**
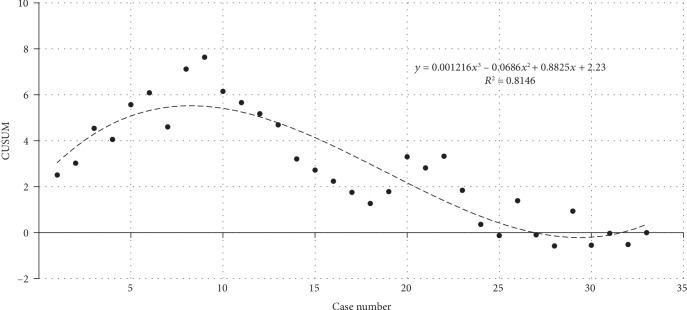
Learning curve for RAG in terms of multidimensional cumulative sum analysis. Dashed line showed the best fitted curve; the equation was *y* = 0.001216*x*^3^–0.0686*x*^2^ + 0.8825*x* + 2.23 (*R*^2^ = 0.8146), where *x* represented the case number. The cut-off value was the 8th case.

In terms of the learning curve of robot-assisted surgery, the operation time of all LAG and RAG cases is reported in Figure 2; the single-dimension CUSUM analysis was used to evaluate the learning curve (Figure 3). According to the equation of the fitted curve in single-dimension CUSUM analysis, we found that the cut-off value was the 10th case in terms of operation time. However, from the multidimensional CUSUM analysis, we found a better result when we set four variables as previously described (Figure 4). The equation of the fitted curve in multidimensional CUSUM analysis showed that the cut-off value was the 8th case with a higher *R*^2^ value as 0.8146, which meant a rapider learning curve.

### 3.5. Subgroup Analysis

For the subgroup analysis, we separated the series into two subgroups according to the cut-off value in multidimensional CUSUM analysis. The first half (FH), which is defined as the initial cases, included 8 RAG cases and 20 LAG cases before the cut-off value of RAG, while the second half (SH), which is defined as the experienced cases, contained 25 RAG cases and 68 LAG cases after the cut-off value of RAG. Characteristics and surgical outcomes are shown in Table 2. We could find that the operation time of RAG in both subgroups was still longer than that of LAG (*p* = 0.014 and *p* = 0.006, respectively). The estimated blood loss of RAG in FH was similar to that of LAG (87.5 ± 51.8 vs. 87.0 ± 22.7, *p* = 0.784), while it was significantly lesser than that of LAG in SH (54.4 ± 34.7 vs. 75.0 ± 34.3, *p* = 0.003). However, robotic surgery got lesser harvested lymph nodes in SH (*p* = 0.008). Meanwhile, we found that the number of retrieved No. 9 LNs in the RAG group was higher than that in the LAG group in SH (3.6 ± 2.3 vs. 2.6 ± 1.7, *p* = 0.045). No significant difference was found in the two subgroups such as harvested suprapancreatic lymph nodes, days of the first passing flatus, hospital stay after surgery, postoperative complications, or rate of 30-day readmission after surgery.

Meanwhile, we compared the differences between FH and SH of RAG. Baseline characteristics were well balanced in the two subgroups, and the operation time of FH was longer than that of SH, although there was no significant difference (368.1 ± 73.2 min vs. 321.9 ± 54.1 min, *p* = 0.063). The estimated blood loss of FH was 87.5 ± 51.8 ml while it was 54.4 ± 34.7 ml in SH (*p* = 0.074). The number of harvested No. 9 LNs was similar in both subgroups (2.4 ± 1.1 vs. 3.6 ± 2.3, *p* = 0.199); the incidence of postoperative complications in SH was significantly lower than that in FH (50.00% vs. 8.00%, *p* = 0.020). No significant differences were found in other parameters. The results are shown in [Supplementary-material supplementary-material-1].

## 4. Discussion

There were more and more evidences that supported that robot-assisted surgery might have similar safety and efficiency for gastric cancer patients compared with laparoscopic-assisted surgery and open surgery in the era of minimally invasive surgery. Some studies found that RAG had a reduction of estimated blood loss, comparable retrieved LNs, and similar postoperative recovery [17–19]. It had been shown that robotic surgery could be an alternative option for gastric cancer patients.

In our study, compared with laparoscopic-assisted gastrectomy, patients who underwent robotic surgery had a similar result in postoperative short outcomes, including postoperative complications, days of the first passing flatus, and hospital stay after surgery. Yoon et al. also describe the similar results in terms of operative outcomes [20]. For the rate of 30-day readmission after surgery, we found that there was no significant difference between the two groups; it was also suggested that robot-assisted gastrectomy was comparable to laparoscopic-assisted surgery in respect to surgical safety and feasibility. Notwithstanding, some researchers found that robotic surgery has a shorter postoperative hospital stay compared with laparoscopic surgery; one cause may be the younger age and less comorbidity in the robotic surgery group [21, 22].

For lymphadenectomy, the total number of retrieved lymph nodes of the RAG group was fewer than that of the LAG group. This may be explained by the relative early stage of the RAG group, although there were no significant differences between the two groups. Furthermore, our results showed that robot-assisted surgery might have its own advantage in the dissection of No. 9 lymph nodes. And subgroup analysis pointed out that RAG could harvest more No. 9 lymph nodes in experienced cases. In the process of lymphadenectomy in gastric surgery, one of the severe intraoperative complications was injury of splenic vessels. Compared with laparoscopic surgery, it was convenient for the surgeon to complete lymphadenectomy in the suprapancreatic area with full exposure of splenic vessels by using a robotic surgical system, depending on the technical advantages of the robotic surgical system such as EndoWrist, three-dimensional vision, tremor filtering, and motion scaling. Thus, surgeons might dissect lymph nodes in the suprapancreatic area more effectively and without worry of unexpected vessel injury, attempting to harvest more lymph nodes. In the process of suprapancreatic lymph node dissection, surgeons should compress the pancreas skillfully to get a better surgical field while pancreatic injury usually occurs in this process. Nevertheless, excellent degrees of freedom provided by EndoWrist made surgeons operate in a deeper space, thus avoiding the unnecessary pancreatic compression, making the dissection and suture more accurate and stable. These technical advantages of the robotic surgical system were beneficial to the dissection of suprapancreatic lymph nodes and might reduce the chance of pancreatic injury. Kim et al. also found that robot-assisted distal gastrectomy was favorable in the dissection of lymph nodes in the suprapancreatic area around the splenic vessels [21]; they found that RAG had a favorable tendency in dissecting LNs at the No. 9 station, although there was no significant difference. And Seo et al. had reported the advantage of robot-assisted distal gastrectomy in terms of postoperative pancreatic fistula incidence [23].

Our study found that the RAG group had a longer operation time compared with the LAG group; it was due to the setting and docking time, slow motion, and swap of robotic arms in the da Vinci robotic system. Meanwhile, the learning curve of robotic surgery might result in longer operation time. It also brings up some characteristics of the learning curve of robotic surgery. From our study, we got a rapid learning curve based on a wealth of experience in laparoscopic gastrectomy. It was less than what some studies have reported [24, 25], which also suggested that surgeons can rapidly adapt the operation of the robotic surgical system with experience of laparoscopic surgery or initial robotic surgery [26–28]. This rapid learning curve might be due to the similar operative environment and procedure compared with laparoscopic surgery. However, the operation time of RAG was still longer than that of LAG after overcoming the learning curve. Moreover, we found that the estimated blood loss in the RAG group was significantly lower than that in the LAG group; this result was similar to previous studies [29, 30].

Furthermore, the results of subgroup analysis showed that the decreased blood loss may be explained by overcoming the learning curve. Zhou et al. also reported that lower EBL was found in experienced cases compared with initial cases [31]. On the other hand, the da Vinci robotic system can filtrate the tremor of hands and provide a three-dimensional vision instead of a two-dimensional vision, and the function of the action scale setting improves the accuracy and stability of surgery, while the internal articulated EndoWrist allows seven degrees of freedom for dissection and suture. These advantages of robotic surgery may be beneficial for reducing blood loss of gastric cancer patients.

Our study also had seldom limitations. First, this study is a retrospective study so that it may consist of some selection bias. Second, survival condition was not evaluated as a prognosis factor in these patients; this study only examined the surgical and postoperative outcomes. Third, the sample size in our study is relatively small so that the statistical analysis may have type II errors. Therefore, the prospective, large-scaled, randomized controlled studies to compare the long-term oncological prognosis between RAG and LAG were appealed.

## 5. Conclusion

In conclusion, the robot-assisted gastrectomy is safe and feasible for gastric cancer patients. It has superiority in the dissection of No. 9 lymph nodes. However, long-term outcomes of RAG by randomized clinical trials are warranted for further investigation.

## Figures and Tables

**Table 1:** Clinicopathological characteristics of all patients.

Clinicopathological features	LAG (*N* = 88)	RAG (*N* = 33)	*p* value
Gender (male/female)	65/23	24/9	0.900
Age (years)	54.7 ± 10.9	55.6 ± 10.3	0.678
ASA (2/3)	73/15	30/3	0.419
BMI (kg/m^2^)	22.59 ± 2.95	22.38 ± 3.03	0.725
Comorbidity	21 (23.86%)	7 (21.21%)	0.758
Resection type (total/distal)	25/63	7/26	0.424
Reconstruction type (B-I/B-II/R-Y)	19/44/25	10/15/8	0.602
Tumor location (U/M/L)	16/15/57	7/5/21	0.917
Operation time (min)	290.6 ± 39.0	333.1 ± 61.4	**0.001** ^∗^
Estimated blood loss (ml)	77.7 ± 32.3	62.4 ± 41.2	**0.005** ^∗^
Tumor size (cm)	2.9 ± 1.3	2.3 ± 1.1	**0.036** ^∗^
T stage			0.075
T1a	18 (20.45%)	13 (39.39%)	
T1b	19 (21.59%)	5 (15.15%)	
T2	20 (22.73%)	10 (30.30%)	
T3	23 (26.14%)	5 (15.15%)	
T4a	8 (9.09%)	0 (0.00%)	
N stage			0.636
N0	47 (53.41%)	23 (69.70%)	
N1	17 (19.32%)	4 (12.12%)	
N2	14 (15.91%)	3 (9.09%)	
N3a	9 (10.23%)	3 (9.09%)	
N3b	1 (1.14%)	0 (0.00%)	
TNM stage			0.168
Ia	30 (34.09%)	16 (48.48%)	
Ib	13 (14.77%)	6 (18.18%)	
IIa	17 (19.32%)	4 (12.12%)	
IIb	7 (7.95%)	5 (15.15%)	
IIIa	11 (12.50%)	0 (0.00%)	
IIIb	7 (7.95%)	2 (6.06%)	
IIIc	3 (3.41%)	0 (0.00%)	
Number of harvested LNs	37.4 ± 13.7	30.3 ± 10.2	**0.008** ^∗^
Number of harvested No. 9 LNs	2.5 ± 1.6	3.4 ± 2.1	**0.039** ^∗^
Number of harvested suprapancreatic LNs	10.3 ± 4.2	10.9 ± 4.8	0.472
Days of the first passing flatus (days)	4.2 ± 1.2	4.8 ± 1.4	0.053
Hospital stay after surgery (days)	8.8 ± 3.3	8.8 ± 2.8	0.579
Postoperative complications	12 (13.64%)	6 (18.18%)	0.735
30-day readmission after surgery	2 (2.27%)	1 (3.03%)	1.000

^∗^ *p* < 0.05, statistical significance.

**Table 2:** Comparison of two subgroups according to the learning curve of the RAG group.

Clinicopathological features	FH (*N* = 28)		*p* value	SH (*N* = 93)		*p* value
	LAG = 20	RAG = 8		LAG = 68	RAG = 25	
Gender (male/female)	17/3	5/3	0.311	48/20	19/6	0.606
Age (years)	56.2 ± 10.2	56.9 ± 11.3	0.870	54.3 ± 11.1	55.2 ± 10.2	0.715
ASA (2/3)	15/5	6/2	1.000	58/10	24/1	0.291
BMI (kg/m^2^)	22.61 ± 3.42	22.00 ± 3.47	0.673	22.59 ± 2.82	22.50 ± 2.94	0.897
Comorbidity	2 (10.00%)	1 (12.50%)	1.000	19 (27.94%)	6 (24.00%)	0.704
Resection type (total/distal)	5/15	3/5	0.651	20/48	4/21	0.190
Reconstruction type (B-I/B-II/R-Y)	5/10/5	2/3/3	0.865	14/34/20	8/12/5	0.444
Tumor location (U/M/L)	3/2/15	3/2/3	0.142	13/13/42	4/3/18	0.744
Operation time (min)	283.7 ± 35.5	368.1 ± 73.2	**0.014** ^∗^	292.7 ± 40.0	321.9 ± 54.1	**0.006**
Estimated blood loss (ml)	87.0 ± 22.7	87.5 ± 51.8	0.784	75.0 ± 34.3	54.4 ± 34.7	**0.003** ^∗^
Tumor size (cm)	2.9 ± 1.2	2.1 ± 1.3	0.147	2.8 ± 1.3	2.4 ± 1.0	0.121
T stage			1.000			0.065
T1a	4 (20.00%)	2 (25.00%)		14 (20.59%)	11 (44.00%)	
T1b	3 (15.00%)	1 (12.50%)		16 (23.53%)	4 (16.00%)	
T2	7 (35.00%)	3 (37.50%)		13 (19.12%)	7 (28.00%)	
T3	5 (25.00%)	2 (25.00%)		18 (26.47%)	3 (12.00%)	
T4a	1 (5.00%)	0 (0.00%)		7 (10.29%)	0 (0.00%)	
N stage			0.685			0.550
N0	11 (55.00%)	5 (62.50%)		36 (52.94%)	18 (72.00%)	
N1	3 (15.00%)	2 (25.00%)		14 (20.59%)	2 (8.00%)	
N2	4 (20.00%)	0 (0.00%)		10 (14.71%)	3 (12.00%)	
N3a	2 (10.00%)	1 (12.50%)		7 (10.29%)	2 (8.00%)	
N3b	0 (0.00%)	0 (0.00%)		1 (1.47%)	0 (0.00%)	
TNM stage			0.951			0.270
Ia	6 (30.00%)	3 (37.50%)		24 (35.29%)	13 (52.00%)	
Ib	3 (15.00%)	2 (25.00%)		10 (14.71%)	4 (16.00%)	
IIa	5 (25.00%)	1 (12.50%)		12 (17.65%)	3 (12.00%)	
IIb	2 (10.00%)	1 (12.50%)		5 (7.35%)	4 (16.00%)	
IIIa	2 (10.00%)	0 (0.00%)		9 (13.24%)	0 (0.00%)	
IIIb	2 (10.00%)	1 (12.50%)		5 (7.35%)	1 (4.00%)	
IIIc	0 (0.00%)	0 (0.00%)		3 (4.41%)	0 (0.00%)	
Number of harvested LNs	34.5 ± 13.0	31.5 ± 6.9	0.444	38.3 ± 13.9	29.9 ± 11.1	**0.008** ^∗^
Number of harvested No. 9 LNs	2.3 ± 1.3	2.4 ± 1.1	0.663	2.6 ± 1.7	3.6 ± 2.3	**0.045** ^∗^
Number of harvested suprapancreatic LNs	9.1 ± 4.2	8.9 ± 2.6	0.914	10.6 ± 4.2	11.6 ± 5.2	0.372
Days of the first passing flatus (days)	4.9 ± 1.0	5.4 ± 1.3	0.395	4.0 ± 1.2	4.6 ± 1.4	0.092
Hospital stay after surgery (days)	10.6 ± 5.1	10.6 ± 4.4	0.656	8.2 ± 2.3	8.2 ± 1.9	0.728
Postoperative complications	4 (20.00%)	4 (50.00%)	0.172	8 (11.76%)	2 (8.00%)	0.887
30-day readmission after surgery	0 (0.00%)	0 (0.00%)	1.000	2 (2.94%)	1 (4.00%)	1.000

^∗^ *p* < 0.05, statistical significance.

## Data Availability

The datasets used and/or analyzed during the current study are available from the corresponding author on reasonable request.

## Supplementary Materials

Supplementary Materials Table S1 Comparsion of FH and SH in RAG series according to the learning curve.
