# Supplementary material for: Robot-Assisted versus Laparoscopic-Assisted Gastrectomy among Gastric Cancer Patients: A Retrospective Short-Term Analysis from a Single Institution in China
Source: Gastroenterol Res Pract. 2019 Oct 23;2019:9059176. doi: 10.1155/2019/9059176 (PMC6855037; doi:10.1155/2019/9059176)
Supplement: Supplementary Materials — Table S1 Comparsion of FH and SH in RAG series according to the learning curve. [file 9059176.f1.pdf]

Table S1. Comparison of FH and SH in RAG series

Clinicopathogical Features	FH (N=8)	SH (N=25)	P value
Gender (male/female)	5:3	19:6	0.651
Age (years)	56.9±11.3	55.2±10.2	0.702
ASA (2/3)	6:2	24:1	0.139
BMI (kg/m^2)	22.00±3.47	22.50±2.94	0.689
Comorbidity	1(12.50%)	6(24.00%)	0.652
Resection type (total/distal)	3:5	4:21	0.320
Reconstruction type (B-I/B-II/R-Y)	2:3:3	8:12:5	0.672
Tumor location (U/M/L)	3:2:3	4:3:18	0.168
Operation time (min)	368.1±73.2	321.9±54.1	0.063
Estimated blood loss (ml)	87.5±51.8	54.4±34.7	0.074
Tumor size (cm)	2.1±1.3	2.4±1.0	0.599
T stage			0.788
T1a	2 (25.00%)	11 (44.00%)	
T1b	1 (12.50%)	4 (16.00%)	
T2	3 (37.50%)	7 (28.00%)	
T3	2 (25.00%)	3 (12.00%)	
T4a	0 (0.00%)	0 (0.00%)	
N stage			0.496
N0	5 (62.50%)	18 (72.00%)	
N1	2 (25.00%)	2 (8.00%)	
N2	0 (0.00%)	3 (12.00%)	
N3a	1 (12.50%)	2 (8.00%)	
N3b	0 (0.00%)	0 (0.00%)	
TNM stage			0.860
Ia	3 (37.50%)	13 (52.00%)	
Ib	2 (25.00%)	4 (16.00%)	
IIa	1 (12.50%)	3 (12.00%)	
IIb	1 (12.50%)	4 (16.00%)	
IIIa	0 (0.00%)	0 (0.00%)	
IIIb	1 (12.50%)	1 (4.00%)	
IIIc	0 (0.00%)	0 (0.00%)	
Number of harvested LNs	31.5±6.9	29.9±11.1	0.709
Number of harvested No.9 LNs	2.4±1.1	3.6±2.3	0.199
Number of harvested suprapancreatic LNs	8.9±2.6	11.6±5.2	0.176
Days of first passing flatus (days)	5.4±1.3	4.6±1.4	0.138
Hospital stay after surgery (days)	10.6±4.4	8.2±1.9	0.061
Postoperative complications	4 (50.00%)	2 (8.00%)	**0.020***
30-day readmission after surgery	0 (0.00%)	1 (4.00%)	1.000

*$P < 0.05$, statistical significance
